# Carbon Dots-Based Fluorescence Assay for the Facile and Reliable Detection of Ag^+^ in Natural Water and Serum Samples

**DOI:** 10.3390/molecules28041566

**Published:** 2023-02-06

**Authors:** Yuanping Luo, Chen Cui, Xingshou Zhang, Yuxiang Jiang, Zhuang Xiang, Chunyu Ji, Zhili Peng

**Affiliations:** Yunnan Key Laboratory for Micro/Nano Materials & Technology, School of Materials and Energy, Yunnan University, Kunming 650091, China

**Keywords:** carbon dots, Ag^+^ sensing, fluorescence quenching, serum, limit of detection

## Abstract

In this report, red-emissive carbon dots (C-dots) were facilely prepared from *o*-phenylenediamine via microwave-assisted hydrothermal treatment. The C-dots demonstrated excitation wavelength-independent emission with maximums at 621 nm that could be effectively quenched by Ag^+^ via static quenching. This phenomenon was exploited to establish a sensitive fluorescence assay with a low detection limit (0.37 μM) and wide linear range (0–50 μM). In addition, this assay demonstrated excellent selectivity toward Ag^+^, free from the interference of 16 commonly seen metal ions. Most importantly, the assay demonstrated high reliability toward samples in deionized water, mineral water, lake water, and serum, which could indicate potential applications for Ag^+^ monitoring in complicated natural and biological environments.

## 1. Introduction

The pollution caused by the excessive discharge of heavy metal ions has become a significant problem for human health and environmental safety [1], attracting great attention from scientists all over the world. Silver and silver-containing compounds have been widely used for sterilization, pharmaceutical, electrical, photography, and so on [2]. With the increasing release of industrial wastes into the environment without proper treatment each year, Ag ions (Ag^+^) have been designated as one of the most toxic categories of heavy metal pollutants [3]. Hence, the detection of Ag^+^ is of great significance to aquatic ecosystems and human health. Currently, various analytical methods have been developed to quantitatively detect Ag^+^; however, owing to the requirement for expensive instruments and complex sample preparation processes, the practical applications of these methods are greatly limited [4]. Fluorescence detection is a relatively new detection method, with the advantages of high sensitivity, simple operation, and good linearity [5,6]. Therefore, many fluorescence-sensing platforms have been developed for the sensitive and selective detection of Ag^+^.

Among the various fluorescence-sensing platforms, carbon dots (C-dots)-based sensing assays have attracted great attention due to their excellent sensing performances, ease of operation, high environmental benignity, as well as low cost [7,8,9]. In the past few years, a series of C-dots derived sensing platforms has been developed for the detection of Ag^+^, and achieved significance (Supplementary Materials Table S1). For instance, the C-dots assay reported by Lu et al. achieved the sensitive detection of Ag^+^ at a low detection limit (LOD) of 0.037 μM [10]. On the other hand, the assay developed by Huang et al. exhibited a considerable linear range for Ag^+^ detection [11]. Furthermore, a double-reference ion-imprinted ratio fluorescent probe achieving Ag^+^ detection with high sensitivity and selectivity has also been reported [12]. However, these assays also faced several challenges. For example, although most studies reported good to excellent LODs, their linear ranges were generally narrow, greatly limiting their practical applications [10,12,13,14,15]. Some studies reported relatively good LODs and linear intervals; however, their practicality for natural water sensing was not demonstrated [11,16,17,18]. Furthermore, far fewer assays have demonstrated the detection of Ag^+^ in biological samples (i.e., serums) [10,11,12,13,14,15,16,17,18,19,20]. Lastly, the sensing mechanisms of most reported studies have not been fully discussed [12,14,16,21,22]. As such, it is still necessary and important to develop C-dots sensing assays that can sensitively and selectively detect Ag^+^ in natural water and biological samples, with the sensing mechanism clearly elucidated.

Herein, we report a sensitive and selective assay derived from C-dots for the detection of Ag^+^ in both natural water and biological samples. Specifically, the C-dots used in this study were prepared from *o*-phenylenediamine (*o*-PD) via a combination of microwave and hydrothermal treatment. The photoluminescence (PL) of the assay could be effectively quenched by Ag^+^; the degree of PL quenching and the Ag^+^ concentration were linearly related. Exploiting this phenomenon, a facile sensing assay was constructed, which achieved the sensitive detection of Ag^+^ (LOD = 0.37 μM) with a broad linear range (0–50 μM). In addition, the assay also demonstrated high selectivity toward Ag^+^, free from the interference of 16 common ions. Furthermore, the assay was tested for the detection of Ag^+^ in real samples (i.e., mineral water, lake water, and serum) with excellent accuracy, indicating the high practicality of this assay. The sensing of Ag^+^ was mainly via static quenching of C-dots PL after careful investigation of the mechanism.

## 2. Results and Discussion

### 2.1. Synthesis and Characterizations of C-dots

**Figure 1:**
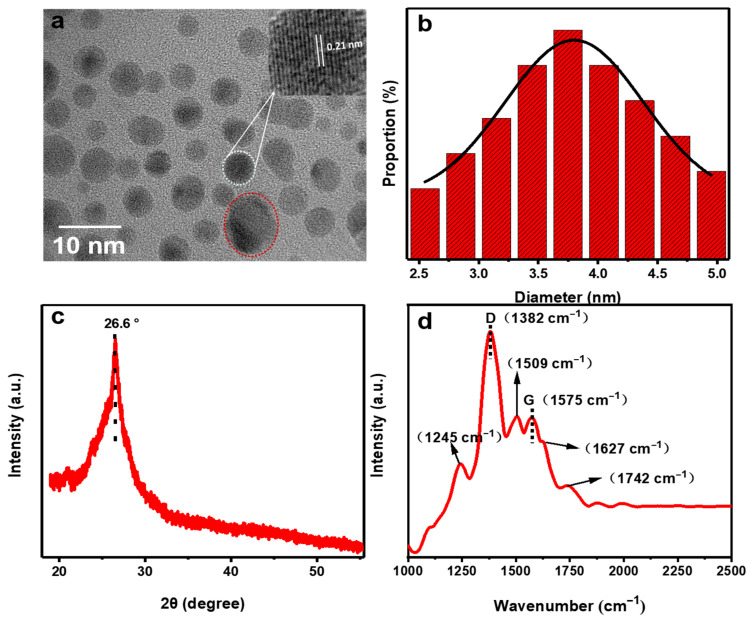
(**a**) TEM images of the C-dots—the upper right inset is an enlarged picture of a specific particle and the particles in the red dashed circles in the lower right corner of the figure may be due to the superposition of several isolated C-dots nanoparticles; (**b**) grain size distribution of C-dots, the black curve represents the fit of the cumulative frequency curve, each legend from left to right shows the proportions of C-dots with diameters of 2.55, 2.85, 3.15, 3.45, 3.75, 4.05, 4.35, 4.65 and 4.95 nm to the total number of selected C-dots; (**c**) XRD spectrum of C-dots, the black dashed line notes the position of the 26.6° peak; (**d**) Raman spectrum of C-dots, in which both characteristic graphitic peaks (D and G bands) and other bands representing polymeric materials are present, the black dashed line notes the positions of the respective peaks.

In this study, black powder C-dots were obtained from *o*-PD via microwave-assisted hydrothermal treatment. The morphological, structural, optical, and chemical properties of the sample were then systematically characterized by TEM, XRD, Raman spectroscopy, UV-vis, fluorescence emission spectroscopy, FTIR, and XPS. Firstly, the morphology of the obtained C-dots was analyzed using TEM. It could be seen that the majority of the C-dots were well dispersed, without obvious aggregation, with diameters ranging from 2.5 to 5.0 nm (Figure 1a and Figure S1); according to the histogram, the average diameter was 3.8 nm (Figure 1b). It is worth mentioning that there were a few particles with diameters near to or exceeding 10 nm, which might be attributed to the stacking of several individual C-dots nanoparticles (particles in the red dotted circle, Figure 1a. The HRTEM showed an obvious lattice fringe with a lattice spacing distance of 0.21 nm (Figure 1a, inset), which could be attributed to the (100) in-plane lattice of graphene [23,24].

In order to further study the structural characteristics, the C-dots were characterized by XRD. As can be seen (Figure 1c), the XRD pattern showed a broad diffraction peak at 26.6°, which might be related to the (002) plane of graphite. This type of broad peak was commonly reported in other studies and was often a sign of successful C-dot preparation [25,26,27,28]. In addition, the intrinsic structure of C-dots was characterized by Raman spectroscopy (Figure 1d). In the 1000–2000 cm^−1^ region, the characteristic D (1382 cm^−1^) and G bands (1575 cm^−1^), as well as some other bands with polymeric characteristics, were observed in the Raman spectra, indicating that they were not typical graphite derivatives [29]. It was noticeable that there was another peak of the same intensity (1509 cm^−1^) next to the G peak, probably due to a perturbation caused by the strong fluorescence peak of the sample [26]. While the peak at 1245 cm^−1^ could be assigned to the D** peak, the peak at 1627 cm^−1^ could be assigned to the D’ peak, demonstrating a highly disordered structural feature of the obtained material [30]. Moreover, the intensity ratio of I_D_/I_G_ was 1.97, which again indicated the presence of a high degree of disorderliness in the sample.

**Figure 2:**
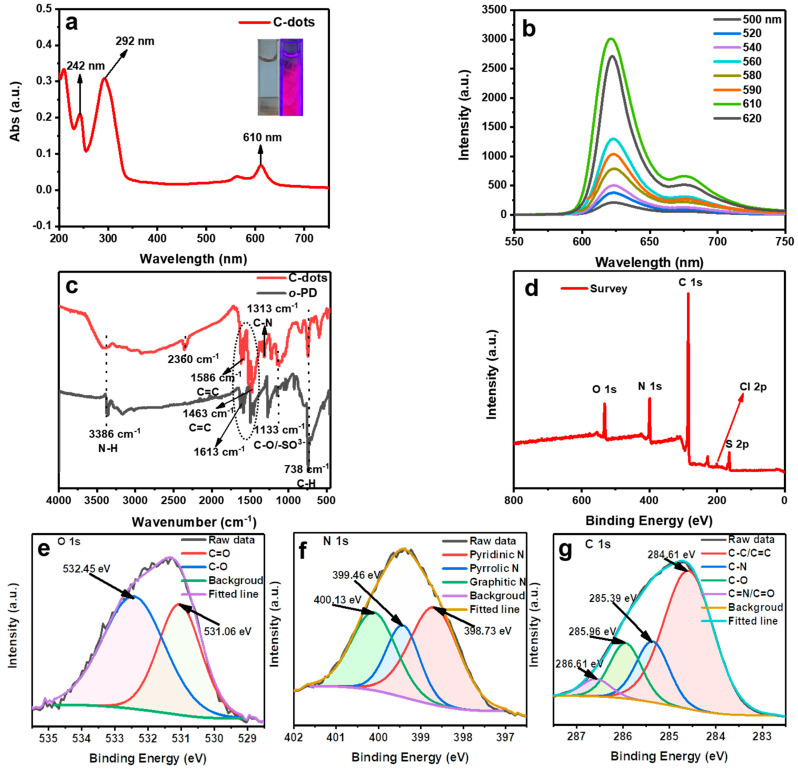
(**a**) UV-vis absorption spectra of C-dots; the left and right insets are pictures of the C-dots dispersion under ambient light and UV-light excitation, respectively. (**b**) Fluorescence emission spectra of the aqueous C-dots dispersion of 17.2 mg/L. (**c**) FTIR spectra of the raw material *o*-PD (black) and C-dots (red), respectively. (**d**) XPS survey spectrum of C-dots showing the presence of O, N, C, Cl, and S elements. High-resolution XPS spectra of C-dots showing O 1s (**e**), N 1s (**f**), and C 1s (**g**), respectively.

To further evaluate the optical behaviors of the obtained samples, UV-vis and fluorescent spectroscopy were carried out. As shown, the C-dots dispersion was transparent, light blue under ambient light (Figure 2a, left inset), and turned red upon excitation by a 400 nm handheld UV lamp (Figure 2a, right inset). The UV-vis absorption of C-dots exhibited two absorption bands at 242 and 292 nm (Figure 2a), which could be attributed to the π-π^*^ transition of C = C and the n-π^*^ transition of C = O/C = N, respectively [24]; the peaks above 400 nm could be attributed to core absorption of C-dots. In addition, unlike most reported C-dots, the fluorescence spectra showed that the emissions of C-dots were independent of the excitation wavelength. Specifically, regardless of the shift in the excitation wavelength, the emission always presented the main peaks at 621 nm and shoulder peaks at 675 nm (Figure 2b) [31].

To study the chemical composition and functionalities of the C-dots, the sample was further characterized by FTIR and XPS. In the FTIR spectrum (Figure 2c), FTIR spectra of both the carbon precursor *o*-PD and C-dots were taken to compare their subtle differences. As expected, various functional groups, such as N–H (3386 cm^−1^), C=C (1620 and 1463 cm^−1^), and C–H (738 cm^−1^) present in *o*-PD were also observed in the C-dots, indicating that the C-dots indeed inherited the distinctive features of their precursor *o*-PD. Of course, the C-dots did exhibit their own characteristic absorption bands due to polymerization and carbonization. For example, the absorption band at 2360 cm^−1^ could be attributed to S–H stretching vibration, the absorption peak at 1613 cm^−1^ could be attributed to the C=O bond, and the peak at 1133 cm^−1^ could be attributed to C–O/–SO^3-^ bonds [32]. 

In the full-scan XPS survey, five peaks at 533.70, 400.91, 283.77, 198.22, and 163.43 eV were observed, corresponding to O 1s, N 1s, C 1s, Cl 2p, and S 2p, respectively (Figure 2d). The high-resolution spectrum of O 1s showed that oxygen existed in the form of C=O (531.06 eV) and C–O (532.45 eV) (Figure 2e). The high-resolution N 1s spectrum displayed pyridinic, pyrrolic, and graphitic N at 398.73, 399.46, and 400.13 eV, respectively (Figure 2f). In the C 1s high-resolution XPS spectrum (Figure 2g), four distinct peaks at 284.61, 285.39, 285.96, and 286.61 eV, corresponding to C–C/C=C, C–N, C–O, and C=N/C=O, respectively, could be observed [33]. In the Cl 2p spectrum [34,35] (Figure S2), the peak at 200.60 eV was ascribed to Cl 2p and the peak at 202.15 eV corresponded to Cl 2p_1/2_. In the S 2p spectrum (Figure S3), the two peaks at 163.68 and 168.24 eV indicated the presence of two forms of sulfur. The former peak could be decomposed into two different components at 163.61 and 164.74 eV, corresponding to 2p_3/2_ and 2p_1/2_. The latter peak was divided into three different components at 167.50, 168.39, and 169.25 eV, all of which were related to sulfur oxides [32]. In general, the XPS results were highly consistent with the FTIR characterizations.

### 2.2. Development of Sensing Assay

**Figure 3:**
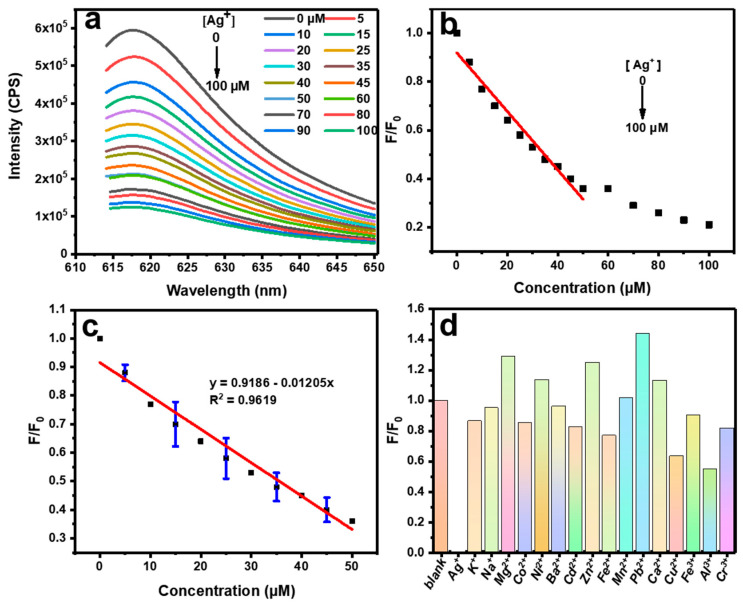
(**a**) Fluorescence emission spectra of C-dots in the presence of Ag^+^ at different concentrations (0–100 μM), the different color curves from top to bottom represent different Ag^+^ concentrations. (**b**) Relationship between F/F_0_ and Ag^+^ concentration from 0–100 μM, where F and F_0_ are the emission intensities of C-dots at 621 nm in the presence and absence of Ag^+^, respectively; the red line passes through the linear region (0–50 μM) of the curve, the black dots represent the fluorescence ratios corresponding to each Ag^+^ concentration. (**c**) Established calibration curve showing the linear detection range of Ag^+^ from 0 to 50 μM, and the blue lines are the error bars at 5, 15, 25, 35, and 45 μM, the black dots represent the fluorescence ratios corresponding to each Ag^+^ concentration, and the red line represents the linear curve fitted from 0–50 μM. (**d**) Fluorescence ratios of F/F_0_ in the presence of C-dots dispersions (blank) and 16 common metal ions (from left to right, Ag^+^, K^+^, Na^+^, Mg^2+^, Co^2+^, Ni^2+^, Ba^2+^, Cd^2+^, Zn^2+^, Fe^2+^, Mn^2+^, Pb^2+^, Ca^2+^, Cu^2+^, Fe^3+^, Al^3+^ and Cr^3+^), where F and F_0_ are the emission intensities of C-dots at 621 nm in the presence and absence of the respective metal ions, as indicated in the figure.

To our delight, the PL of C-dots could be effectively quenched by Ag^+^, and the degree of quenching was closely correlated with the concentration of Ag^+^. By exploiting this phenomenon, a sensitive and selective fluorescent assay for the detection of Ag^+^ was developed from C-dots. To begin with, we firstly optimized the concentration of C-dots and the incubation time; the optimum concentration of C-dots was determined to be 11.5 mg/L based on their PL intensities (Figure S4a) and the incubation time was set at 10 min (Figure S4b). The relationship between the Ag^+^ concentration and PL intensity of C-dots was then explored based on the optimized conditions. With the increase in the Ag^+^ concentrations from 0 to 100 μM, the emissions of the C-dots dispersion at 621 nm gradually decreased (Figure 3a). To better express the relationship between the Ag^+^ concentration and PL intensity of C-dots, we plotted a scheme with the ratio of fluorescence intensity (F/F_0_) along the *y*-axis and Ag^+^ concentration along the *x*-axis, where F_0_ and F represented the emission intensities of the C-dots dispersions without and with Ag^+^, respectively. As can be clearly seen (Figure 3b), F/F_0_ was linearly related to the Ag^+^ concentration in the range of 0–50 μM and had excellent linearity. Based on this linearity, a calibration curve (Figure 3c) with a linear equation of F/F_0_ = 0.9186–0.01205 [Ag^+^], R^2^ = 0.9619 was established. According to the well-accepted 3σ method [31], the LOD of this assay was determined to be 0.37 μM, which was far below the maximum level of Ag^+^ allowed in drinking water (0.93 μM) as specified by the World Health Organization (WHO) [10].

With the successful establishment of the sensing assay, we then conducted a series of experiments to investigate its selectivity (Figure 3d). It is well known that many ions could coexist in solution; thus, the ability to be free of interference from other metal ions is very important for metal ion sensing. It could be seen that, under the same conditions, common monovalent (i.e., K^+^ and Na^+^), divalent (i.e., Mg^2+^, Co^2+^, Ni^2+^, Ba^2+^, Cd^2+^, Zn^2+^, Fe^2+^, Mn^2+^, Pb^2+^, Ca^2+^, and Cu^2+^), and trivalent metal ions (i.e., Fe^3+^, Al^3+^, and Cr^3+^) did not cause significant decreases in the PL intensities. On the other hand, Ag^+^ could induce a sharp decrease in the PL of C-dots.

### 2.3. Applicability and Reliability of the Assay

To test the reliability of this sensing assay, three spike solutions of Ag^+^ with different concentrations were prepared and the calculated concentrations based on the calibration curve established above (Figure 3c) were compared to the actual concentrations. To our delight, the assay developed in this study appeared to be very reliable for all of the spike solutions, with recoveries ranging from 95.64 to 104.00% (Table 1, rows 1–3). Specifically, when the concentration of the Ag^+^ solution was 40 μM, the recovery was as high as 97.23% (Table 1, row 2).

As the simulated environment in the above experiments was DI water, additional experiments were conducted to explore the practicality of this assay for sensing in real water samples. To begin, the presence of Ag^+^ in mineral water and lake water samples was evaluated using the assay established above. As shown in the figure (Figure S5), the fluorescence intensities of C-dots in mineral water and lake water were almost the same as that of C-dots in DI water, indicating that the presence of Ag^+^ in these two samples was much lower than the LOD of the assay. Therefore, in order to better evaluate the reliability of this assay in real water samples, we prepared three more spike solutions, each with mineral water and lake water samples. The results showed that the recoveries of the mineral water samples ranged from 95.64 to 104.00% (Table 1, rows 4–6), while those of lake water samples ranged from 91.96 to 94.51% (Table 1, rows 7–9), both of which demonstrated excellent recoveries, indicating the high practicability of this assay for Ag^+^ detection in real water environments.

On the basis of the above explorations in real water samples, we further extended the sensing of Ag^+^ to a biological sample with fetal bovine serum. Serum is a classic biological medium containing complex biological substances, such as proteins, antibodies, drugs, etc. Additionally, Ag^+^ in serum could be used as an indicator of metabolic-related diseases [36]. Therefore, four Ag^+^ solutions of different concentrations were prepared with fetal bovine serum, and the concentrations detected by the established calibration curve were again compared to the actual concentrations. To our delight, the recoveries of Ag^+^ in fetal bovine serum were found to be between 93.67 and 104.33% (Table 1, rows 10–13), demonstrating the high reliability of the C-dots-based Ag^+^ sensing platform, even in complex biological substrates.

### 2.4. Sensing Mechanism Investigation

**Figure 4:**
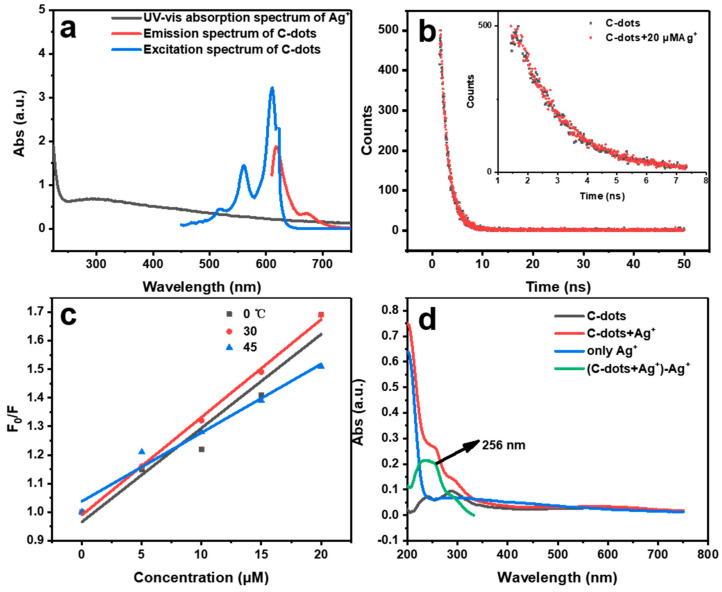
(**a**) UV-vis absorption spectrum of Ag^+^ (black curve), fluorescence emission spectrum of C-dots (red curve), and excitation spectrum of C-dots (blue curve); (**b**) fluorescence decay curves of C-dots in the absence (black) and presence of Ag^+^ (red); the upper-right inset shows the fluorescence decay curves of C-dots between 0.5 and 7.5 ns without Ag^+^ (black) and with Ag^+^ (red); (**c**) comparison of Stern–Volmer equation slopes for Ag^+^ at 0, 30 and 45 °C; (**d**) UV-vis absorption spectra of C-dots only (black curve), C-dots in the presence of Ag^+^ (red curve), Ag^+^ solution (blue curve), and the mathematical curve (green curve) obtained by subtracting the blue curve (Ag^+^ only) from the red curve (C-dots + Ag^+^), respectively. The negative portion of the spectrum in the green curve of the mathematical calculation is not shown, as it has no physical meaning.

The fluorescence quenching of fluorophores or nanoparticles generally falls into four categories, namely dynamic quenching, static quenching, fluorescence resonance energy transfer (FRET), and inner filter effect (IFE) [37]. Among them, FRET refers to fluorescence quenching due to the energy transfer from the fluorophore (donor) to the quencher (acceptor), which requires a certain spectral overlap between the emissions of the donor and the absorptions of the acceptor [38]. Similarly, absorption of the quencher is also required to significantly overlap with the excitation or emission wavelengths of the fluorophores in order for an IFE process to occur [39]. As such, we carefully compared the excitation and emission spectra of C-dots with the absorption spectra of Ag^+^ (Figure 4a). Clearly, there was no significant overlap between the emission (red curve) or excitation spectra (blue curve) of C-dots and the absorption spectrum of Ag^+^ (black curve). These results clearly indicate that the possibility of C-dots emissions quenching by Ag^+^ via FRET and IFE can be largely ruled out.

Dynamic quenching refers to a process in which fluorophores in the excited state return to the ground state without light emission (fluorescence quenching) after colliding with quenchers, which could result in the occurrence of energy transfer or charge transfer. While static quenching is a phenomenon in which the interactions of fluorophores and quenchers could form non-fluorescent, ground-state complexes, and thus quench the fluorescence emissions of fluorophores. As such, the fluorescence lifetimes of fluorophores in typical dynamic quenching vary depending on the presence or absence of the quenchers, and the quenching effect can be greatly enhanced with increased temperature, as the number of collisions between fluorophores and quenchers increases under elevated temperatures. On the other hand, in a typical static quenching, the fluorescence lifetimes of fluorophores stay the same, regardless of the presence or absence of quenchers, and the formation of ground-state complexes could generally lead to changes in the UV-vis absorption of fluorophores. Furthermore, unlike dynamic quenching, increases in temperature generally hinder the formation of ground-state complexes or reduce their stabilities in static quenching.

Thus, we firstly measured the fluorescence lifetimes of C-dots in the absence and presence of Ag^+^ (Figure 4b). Our result showed that the average fluorescence lifetime (τ_0_) of C-dots was 1.66 ns (τ_1_ = 1.25, τ_2_ = 2.01), and with the addition of Ag^+^, the average fluorescence lifetime (τ) of C-dots became 1.64 ns (τ_1_ = 1.64 ns, τ_2_ = 1.64), indicating that the possibility of dynamic quenching could be largely ruled out and the FRET mechanism could also be further ruled out [38]. We then evaluated the temperature effects of the quenching process, as demonstrated, the slopes of the Stern-Vølmer plots stayed similar, despite the significant temperature variations (Figure 4c), indicating that the quenching of the C-dots’ fluorescence emissions was most likely via a static quenching process. Moreover, in general, the Stern-Vølmer quenching constant (Kq) [38] for dynamic quenching should be in the range of (1–2) × 10^10^ L·mol^−1^s^−1^. However, the quenching constant calculated from Figure 3c was 2.04 × 10^13^ L·mol^−1^s^−1^, much higher than the theoretical value allowed for dynamic quenching, again indicating that the quenching of C-dots fluorescence was via a non-kinetic process.

Lastly, we carefully analyzed the interaction of C-dots with Ag^+^ using UV-vis absorption spectra (Figure 4d), which showed the appearance of a new absorption peak at 256 nm for C-dots in the presence of Ag^+^ (green curve vs. black curve). This might be due to the strong coordination between the C=N group on the C-dots and Ag^+^, which was generally considered as a sign of static quenching [40]. Based on the above-mentioned evidence, we cautiously propose that the quenching of C-dots fluorescence by Ag^+^ was mainly through the static quenching path.

## 3. Materials and Methods

### 3.1. Reagents and Materials

AgNO_3_, KCl, NaCl, MgCl_2_, CoCl_2_·6H_2_O, NiCl_2_, BaCl_2_, CdCl_2_, Zn (CH_3_COO)_2_, FeCl_2_, MnCl_2_·4H_2_O, Pb (NO_3_)_2_, CaCl_2_, CuSO_4_, FeCl_3_, AlCl_3_, and CrCl_3_·4H_2_O were purchased from Energy Chemical Reagent Co., Ltd. (Shanghai, China). Sodium hydroxide (NaOH 96%) and hydrochloric acid (HCl 36–38%) were purchased from Kelong Chemical Co., Ltd. (Chengdu, China). *o*-phenylenediamine (*o*-PD) (98%), tertbutyl hydroperoxide (TBHP) (70%), and 3-chloroperbenzoic acid (85%) were obtained from Anhui Zesheng Technology Co., Ltd. (Anqing, China). The deionized (DI) water used in all experiments was produced using a Master Touch-S laboratory ultrapure water machine (Master Touch, Shanghai, China). Mineral water was taken from the laboratory at the Chenggong Campus of Yunnan University; lake water was taken from Ze Lake at the Chenggong Campus of Yunnan University (Kunming, China) and larger particles in the lake water were filtered out with a suction filter funnel before testing. Fetal bovine serum was purchased from Life Technologies (Grand Island, NY, USA) and diluted 100 times with DI water before testing. All the reagents were used as received, without further purification, unless otherwise noted.

### 3.2. Characterization and Instrumentation

A microwave synthesizer (Discover SP of American CEM) with its power, temperature, and pressure set at 200 W, 200 °C, and 250 PSI, respectively, was used for the synthesis of C-dots. Transmission electron microscope (TEM) and high-resolution transmission electron microscope (HRTEM) images of C-dots were obtained using a JEM-2100 transmission electron microscope (Tokyo, Japan), in which the acceleration voltage was 200 kV and the magnification was 800 K times. The TEM test samples were processed as follows: 100 µL of C-dots dispersion (10 mg/L) was dispersed in 20 mL of ethanol and sonicated for 30 min. Then, a drop of the C-dots dispersion was placed on a carbon grid until the ethanol evaporated and TEM tests were performed. The X-ray diffraction (XRD) pattern was obtained on an X-ray diffractometer (DX-2700BH, Haoyuan Instrument Co., Ltd., Dandong, Liaoning, China) with a wavelength (λ) of 0.15406 nm. For XRD sample preparation, appropriate amounts of black powder samples were placed on the sample table and then pressed with a slide to make the samples’ surfaces flat before putting them into the X-ray diffractometer for testing. The Raman spectra of the C-dots were obtained using a micro confocal Raman spectrometer (Raman, inVia, Renishawin, UK).

The absorption spectrum of the C-dots dispersion (17.2 mg/L) was recorded using an ultraviolet-visible spectrophotometer (UV-vis), where the wavelength range was 195–700 nm, the scanning speed was medium, the sampling interval was 1 nm, and the sampling was repeated twice (UV-2600, Shimadzu, Tokyo, Japan). The fluorescence spectrum of C-dots (17.2 mg/L) was tested using a fluorescence spectrometer (FL, F97 Pro, Shanghai Prism Technology Co., Ltd., Shanghai, China). The scanning method was three-dimensional wavelength scanning. The excitation wavelength was 500–750 nm and the excitation width was 10 nm. The emission wavelength was 300–750 nm and the emission width was 10 nm. The scanning speed was 6000 nm/min, the scanning interval was 1 nm, and the gain was 650 V.

The Fourier-transform infrared spectra (FTIR) were recorded from 4000 to 450 cm^−1^ on a Thermo Fisher Scientific NicoletiS50 (Middle Age Walker, Waltham, KS, USA), in which the samples were smeared on KBr before testing. The XPS spectra were obtained directly from the black powder samples using an X-ray photoelectron spectroscope (K-Alpha^+^, Thermo Fisher Scientific, Waltham, MA, USA); the X-ray source was monochromatic Al Kα with a hυ = 1486.6 eV photon energy operated at 6 mA × 12 kV. The frequency-domain lifetime was measured using an FLS10000 fluorescence lifetime spectrometer (Edinburgh, UK).

### 3.3. Preparation of C-Dots

The C-dots were synthesized via the microwave-assisted hydrothermal treatment of *o*-PD, as we previously reported [29]. To be specific, a mixture of *o*-PD (0.25 g), 3-chlorobenzoic acid (0.2 g), and H_2_SO_4_ (2 mL) in DI water (20 mL) was treated with a microwave synthesizer for 20 min. After the reactor was cooled to r.t., the mixed solution was then transferred to a Teflon autoclave and heated at 200 °C for 12 h. After the autoclave was cooled to r.t., the resulting mixture was neutralized with a diluted sodium hydroxide solution and filtered. The residues were then washed with DI water, collected, and dried in a vacuum to obtain the desired product as a black powder.

### 3.4. Sensing Assay Development

Firstly, a C-dots dispersion with a concentration of 17.2 mg/L was prepared and the pH of the dispersion was adjusted to 3. Then, a Ag^+^ mother liquor solution of 3 mM was prepared. Next, the two dispersions/solutions were mixed in different ratios so that the concentrations of C-dots were kept constant (11.5 mg/L) while the concentrations of Ag^+^ were gradually increased (0, 5, 10, 15, 20, 25, 30, 35, 40, 45, 50, 60, 70, 80, 90, and 100 μM). The resulting dispersions were then kept for 10 min before their PL was measured.

### 3.5. Selectivity of the Assay

To investigate the specificity of the sensing system, the interference of other ions, including K^+^, Na^+^, Mg^2+^, Co^2+^, Ni^2+^, Ba^2+^, Cd^2+^, Zn^2+^, Fe^2+^, Mn^2+^, Pb^2+^, Ca^2+^, Cu^2+^, Fe^3+^, Al^3+^, and Cr^3+^, in the assay was evaluated. All procedures were similar to those discussed above, except that the concentrations of the metal ions were set as a constant (750 μM). All the tests were repeated 3 times and performed at r.t.

### 3.6. Reliability of the Assay

To verify the reliability of the assay, 13 different Ag^+^ spike solutions were prepared using DI water, mineral water, lake water, and fetal bovine serum, respectively, and their concentrations were measured according to the calibration curve established in this study. The detected Ag^+^ concentrations were then compared with the actual concentrations to calculate their recoveries.

## 4. Conclusions

In summary, we reported a facile preparation method of C-dots from *o*-PD via microwave-assisted hydrothermal treatment. The C-dots were transparent and light blue under ambient light and red under excitation by a handheld UV lamp. The C-dots exhibited maximum emissions at 621 nm that were independent of the excitation wavelengths and could be effectively quenched by Ag^+^. A sensing platform derived from the C-dots was then established for the sensitive detection of Ag^+^, and the emission of the C-dots dispersion at 621 nm gradually decreased with the gradual increase in the Ag^+^ concentration, achieving a low LOD (0.37 μM) and a wide linear range (0–50 μM). It is worth mentioning that the assay demonstrated superior selectivity toward Ag^+^ and was free from the interference of 16 ions, including K^+^, Na^+^, Mg^2+^, Co^2+^, Ni^2+^, Ba^2+^, Cd^2+^, Zn^2+^, Fe^2+^, Mn^2+^, Pb^2+^, Ca^2+^, Cu^2+^, Fe^3+^, Al^3+^, and Cr^3+^. Most importantly, the platform was capable of detecting Ag^+^ in natural water (i.e., lake water) and biological samples (i.e., serum) with high accuracy, demonstrating its high potential for Ag^+^ sensing in environmental and biological systems. Lastly, the possibility of FRET and IFE quenching was ruled out by analyzing the characteristics of the excitation and emission spectra of C-dots, as well as the absorption spectrum of Ag^+^. Furthermore, based on the fluorescence lifetime study, temperature effects on the quenching process, and analysis of the UV-vis absorption spectra of the C-dots/Ag^+^ complex, it was shown that the quenching of C-dots’ fluorescence emissions by Ag^+^ was mainly via the static quenching path.

## Figures and Tables

**Table 1:** Results of the Ag^+^ recovery experiments performed in mineral water, lake water, and serum.

	Sample	Ag^+^ Added/μM	Found/μM	Recovery/%
**1**		35	36.40	104.00
**2**	DI water	40	38.89	97.23
**3**		45	43.04	95.64
**4**		35	36.4	104.00
**5**	Mineral water	40	40.55	101.38
**6**		45	43.04	95.64
**7**		35	33.08	94.51
**8**	Lake water	40	37.23	93.08
**9**		45	41.38	91.96
**10**		15	15.65	104.33
**11**	Serum	20	18.97	94.85
**12**		30	28.10	93.67
**13**		40	38.06	95.15

## Data Availability

The data presented in this study are available within the article and supplementary material.

## Supplementary Materials

The following supporting information can be downloaded at: www.mdpi.com/article/10.3390/molecules28041566/s1, Table S1: Representative assays derived from C-dots realizing the detection of Ag^+^; Figure S1: TEM images of C-dots with different scale bars; Figure S2: High-resolution XPS spectra of C-dots showing Cl 2p; Figure S3: High-resolution XPS spectra of C-dots showing S 2p; Figure S4: Figures showing sensing parameters optimization; Figure S5: The normalized fluorescence intensities of C-dots dispersions in DI water, mineral water and lake water, respectively.

## Sample Availability

Samples of the C-dots are available from the authors.
