# Supplementary material for: Carbon Dots-Based Fluorescence Assay for the Facile and Reliable Detection of Ag+ in Natural Water and Serum Samples
Source: Molecules. 2023 Feb 6;28(4):1566. doi: 10.3390/molecules28041566 (PMC9963176; doi:10.3390/molecules28041566)
Supplement: Supplementary file 1 [file molecules-28-01566-s001.zip › molecules-2192495-supplementary.pdf]

Supplementary Materials

Carbon Dots-Based Fluorescence Assay for the Facile and Reliable Detection of Ag^{+} in Natural Water and Serum Samples

Yuanping Luo,[a] Chen Cui,[a] Xingshou Zhang,[a] Yuxiang Jiang,[a] Zhuang Xiang,[a] Chunyu Ji,[a] and Zhili Peng[a,*]

[a]Yunnan Key Laboratory for Micro/Nano Materials & Technology, School of Materials and Energy, Yunnan University, Kunming 650091, China.

***Corresponding authors:**

(Z. P.) Tel.: +86–871–65037399; E–mail: zhilip@ynu.edu.cn

Table S1. Representative assays derived from C-dots realizing the detection of Ag^+.

	Carbon precursor [a)]	LOD(μM)	Linear range (μM)	Mechanism Study	Natural water sample testing	Biological samples	Ref [b)].
**1**	bean pod, onion	0.037	0.1-25	Yes	Yes	-	10
**2**	CA, PD	0.031	1-4	Yes	Yes	-	13
**3**	Citric acid, amino acid	0.05	0.1-4	-	Yes	-	14
**4**	ACT, 3-APA	0.00903	0.99-26.04	Yes	Yes	-	15
**5**	3-ABA, 2,5-DA	0.35	1-300	Yes	-	-	11
**6**	4-Bromo aniline, EDA	3.9	0-100	-	-	-	16
**7**	AL, melamine	0.289	0-50	Yes	-	-	17
**8**	GSH	-	0-50	Yes	-	-	18
**9**	GSH,	0.124	0-100	Yes	Yes	-	19
**10**	carrageenan Sodium, LP	0.5	5-290	Yes	Yes	-	20
**11**	Gelatin, 3-ABA	0.086	0.2-12.5	-	Yes	-	12
**12**	CA, VB_1	0.4	0-250	-	Yes	Yes	21
**13**	GSH	6.39	2.5-75	-	Yes	Yes	22
**14**	*o*-PD	0.37	0-50	Yes	Yes	Yes	**This Work**

[a)] CA: anhydrous citric acid; PD: 2,3-phenazinediamine; ACT: ammonium citrate tribasic; 3-APA: 3-aminophenylboronic acid; 3-ABA: 3-aminobenzeneboronic acid; 2,5-DA: 2,5-diaminobenzenesulfonic acid; EDA: ethylenediamine; AL: m-phenylenediamine, p-phenylenediamine, o-phenylenediamine; LP: lignosulphonate; VB_1: Vitamin B1.

[b)] The numbering of the references in the table is in consistent with the main text.

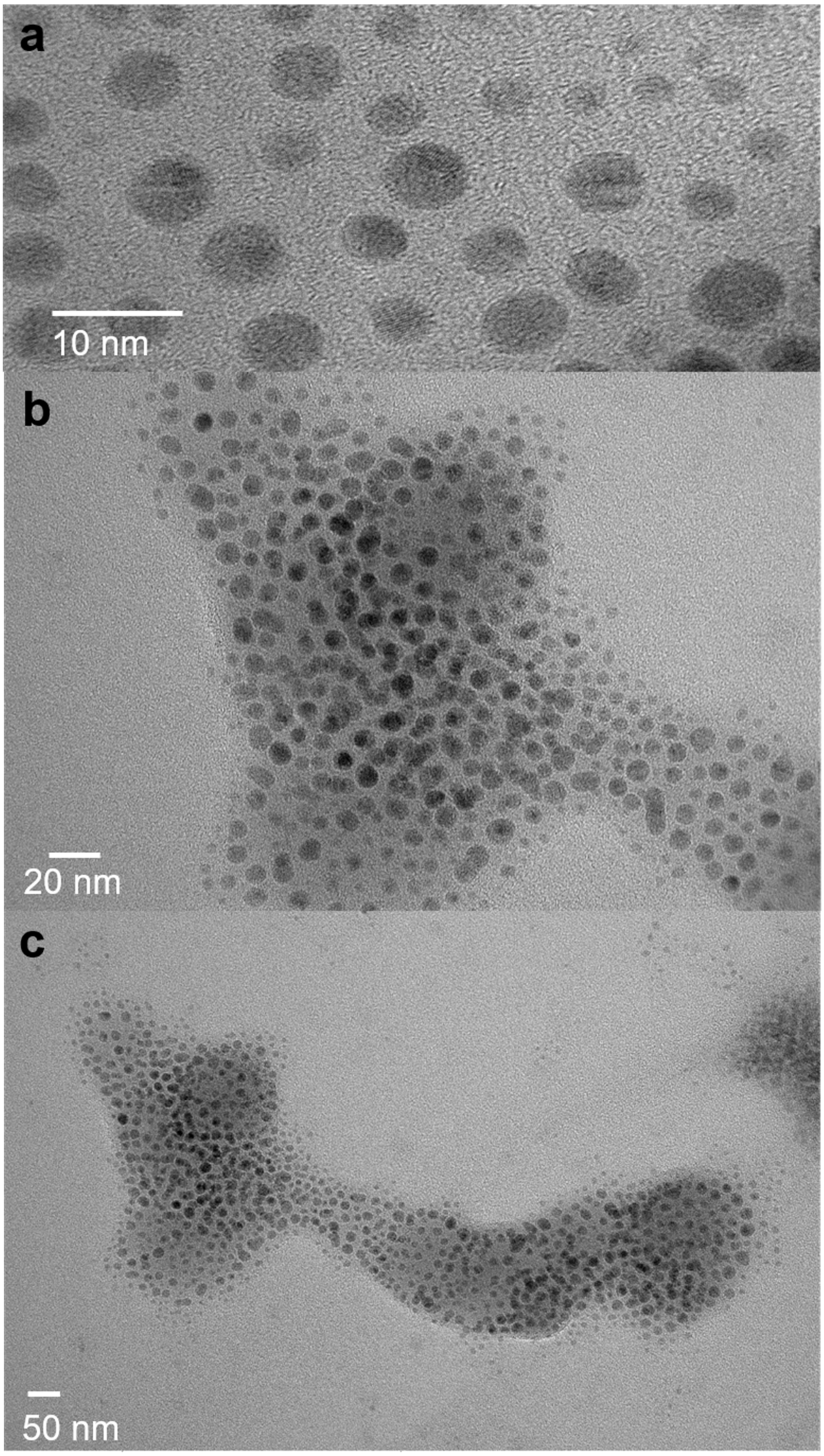

Figure S1. TEM images of C-dots with different scale bars: (**a**) 10 nm, (**b**) 20 nm and (**c**) 50 nm.

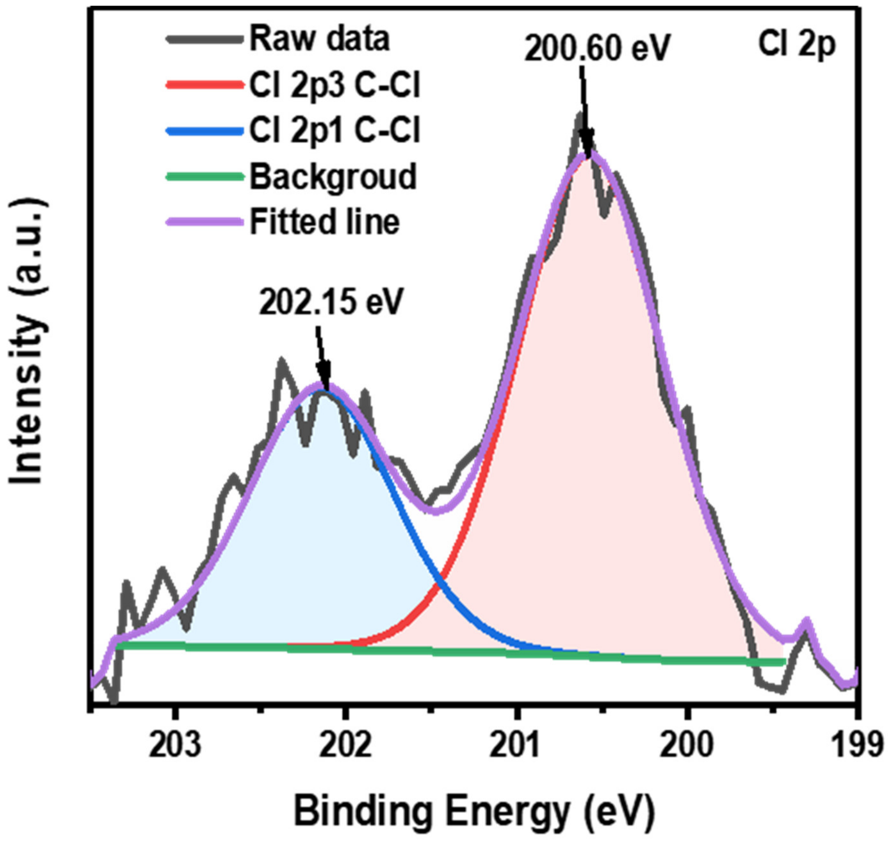

Figure S2. High-resolution XPS spectra of C-dots showing Cl 2p.

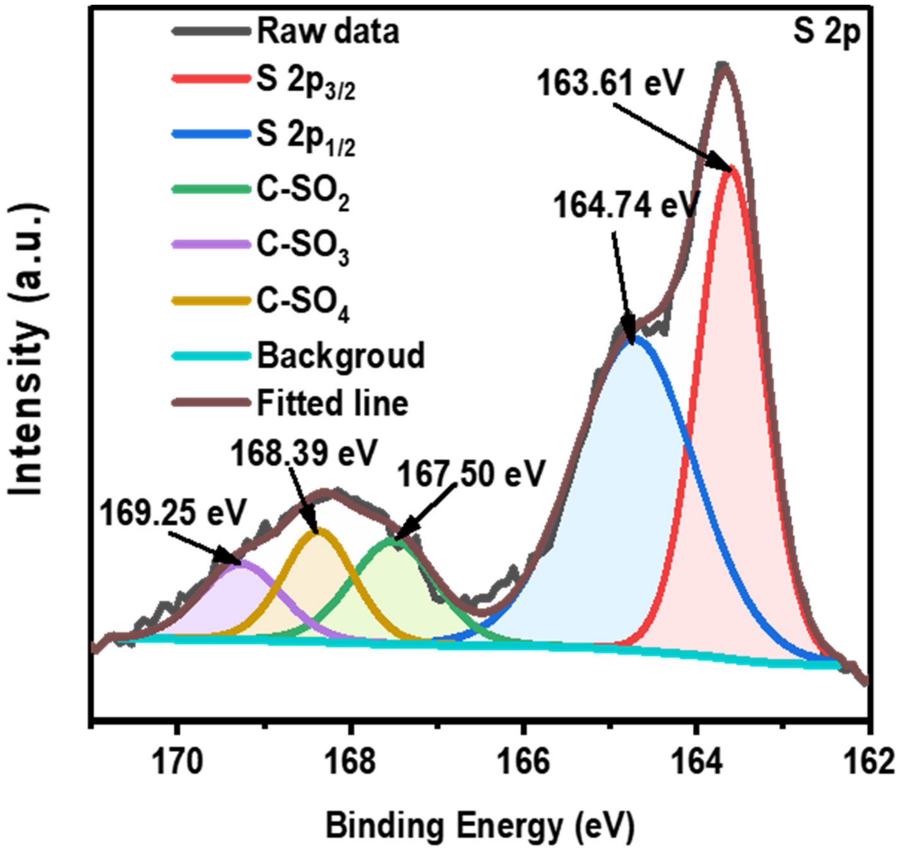

Figure S3. High-resolution XPS spectra of C-dots showing S 2p.

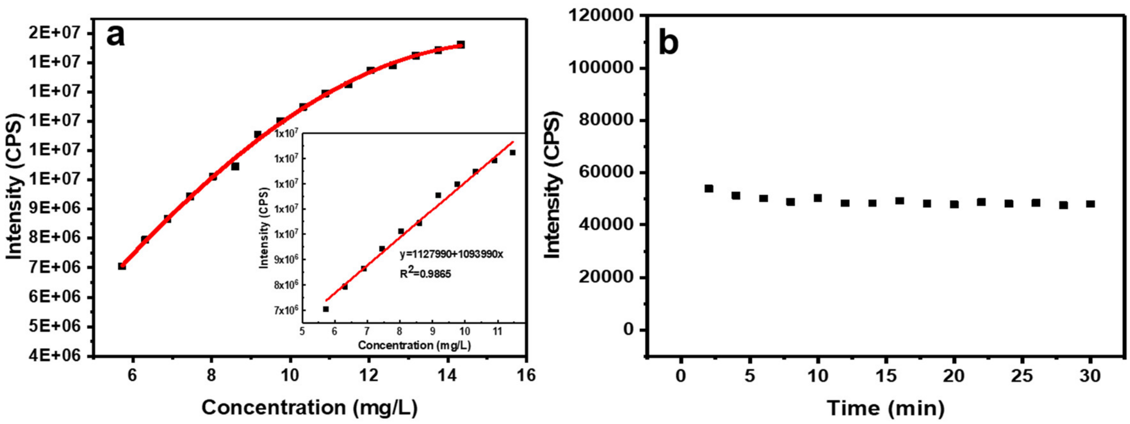

Figure S4. Sensing parameters optimization, (**a**) C-dots concentration optimization; (**b**) incubation time optimization.

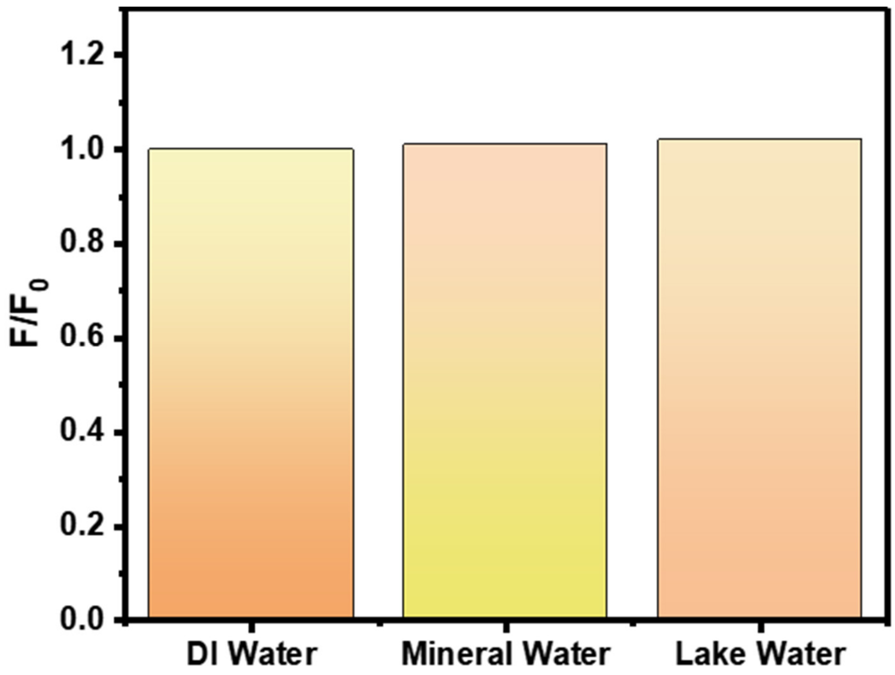

Figure S5. The normalized fluorescence intensities of C-dots dispersions in DI water, mineral water and lake water, respectively.
